# Association of IL-4 with pachychoroid neovasculopathy

**DOI:** 10.1038/s41598-023-28108-y

**Published:** 2023-01-20

**Authors:** Takashi Baba, Ayumi Koyama, Ryu Uotani, Hitomi Miyake, Kodai Inata, Shin-ichi Sasaki, Yumiko Shimizu, Yoshitsugu Inoue, Kaori Adachi, Eiji Nanba, Dai Miyazaki

**Affiliations:** 1 Division of Ophthalmology and Visual Science, Faculty of Medicine, Tottori University, 36-1 Nishicho, Yonago, Tottori 683-8504 Japan; 2 Research Initiative Center, Organization for Research Initiative and Promotion, Tottori University, Yonago, Tottori Japan; 3 Otani Hospital, Tsuyama, Okayama Japan

**Keywords:** Macular degeneration, Chronic inflammation

## Abstract

The purpose of this study was to identify the inflammatory cytokines that were associated with pachychoroid neovasculopathy (PNV). Seventy-five eyes of 75 patients with PNV, 145 eyes of 145 patients with neovascular age-related macular degeneration without pachyvessels, and 150 eyes of 150 normal subjects were examined for the levels of intraocular cytokines. In eyes with PNV, the levels of IL-1α, IL-1β, IL-2, IL-4, IL-10, and VEGF were significantly higher than that of the controls. Logistic regression analysis showed that the highest association with the pachyvessels was found for IL-4, IL-2, and IL-1α. In eyes with PNV, the levels of IL-4, IL-2, IL-5, IL-13, IL-1α, and IL-1β were significantly higher in eyes with both increased choroidal thickness and choroidal vessel diameter. The strongest correlation with the choroidal thickness and vessel diameter was observed for IL-4. In PNV eyes with polypoidal lesions, the levels of IL-4, IL-17, and TNFβ were significantly correlated with the number of polypoidal lesions. Of these cytokines, IL-4 was especially associated with the thickness of the choroidal vessels and the formation of polypoidal lesions. We conclude that IL-4 is most likely involved in establishing the clinical characteristics of PNV and polypoidal vascular remodeling.

## Introduction

Neovascular age-related macular degeneration (nAMD) is a leading cause of severe reduction of vision in the elderly throughout the world. Pachychoroid neovasculopathy (PNV) has been recently proposed as a new disease entity related to nAMD, and it is characterized by the presence of pachyvessels which are dilated choroidal vessels with hyperpermeability^1^. However, the PNV spectrum of diseases has not been examined in detail.

PNV is characterized by the presence of choroidal pachyvessels. The main mechanism causing the pachyvessels is an elevation of the venous pressure with choroidal congestion and intervortex venous anastomoses^2^. The dilation of the choroidal veins causes a reduction of the viscoelasticity of the vessel walls. It has been proposed that pachyvessels are caused by damaged venous outflow in the choroid and elevated outflow pressure caused by congestion. The clinical entities associated with pachyvessels include central serous chorioretinopathy, peripapillary pachychoroid syndrome, and the PNV spectrum of diseases.

The elevated venous pressure and congestion leads to an induction of matrix metalloproteinases, tissue remodeling, and inflammatory cell recruitment. In addition, there is an induction of chemokines including CCL2 and IL-8 and the recruitment of Th1 or Th2 lymphocytes. These are manifested as organ or disease specific characteristics^3^. Thus, venous outflow problems appear to cause a remodeling of the vasculature with a signature of release of inflammatory cytokines or chemokines^4^.

nAMD is known to be associated with a number of intraocular cytokines including VEGF, CCL2^5,6^, IL-6^7–9^, and IL-8^7,9,10^, however information on the cytokines associated with PNV is still limited. This prevents a detailed understanding of the molecular pathology of PNV and thus, the development of therapeutic strategies.

In contrast, PCV, which is often associated with PNV, has been extensively studied because of its association with inflammatory cytokines. For example, PCV was shown to be associated with the levels of IL-4, IL-10, and IL-23 and with exudative lesions in the fluorescein angiograms^11^.

Interestingly, the contribution of the different kinds of cytokines appears to be different for the PNV spectrum of diseases. For example, PNV patients are refractory to standard anti-VEGF therapy, while polypoidal choroidal vasculopathy (PCV) patients generally respond well to anti-VEGF therapy^12^.

Generally, vascular remodeling requires the recruitment of progenitors of endothelial cells, and this is also required for tissue neovascularization. We have shown that IL-4 contributed to choroidal neovascularization by a remodeling process^13^. Thus, IL-4 may also play roles in the vascular remodeling processes seen in PNV. However, this has not been definitively determined.

Thus, the purpose of this study was to determine which inflammatory cytokines are significantly correlated with the PNV spectrum of diseases. To determine this, we used proteomic profiling of the aqueous humor of patients with PNV spectrum of diseases. We also examined how the disease characteristics and related vascular abnormalities were associated with the induced cytokines. We shall show that IL-4 is significantly associated with presence of pachyvessels and the development of polypoidal lesions.

## Results

### Association of inflammatory cytokines with pachychoroid neovasculopathy (PNV) and with neovascular age-related macular degeneration (nAMD) without pachyvessels

The levels of the intracameral cytokines were determined in 75 eyes of 75 patients with PNV, 145 eyes of 145 patients with nAMD without pachyvessels including 93 eyes of 93 patients with polypoidal choroidal vasculopathy (PCV), and 52 eyes of 52 patients with typical AMD without polypoidal lesions. As control, the levels of intracameral cytokines of 150 eyes of 150 normal subjects who underwent cataract surgery were also determined.

The mean age of the PNV patients was 71.3 ± 1.2 years, of the PCV patients was 74.3 ± 0.9 years, and of the typical AMD patients was 75.7 ± 1.2 years (Table 1). The mean age of the control subjects were 73.9 ± 0.6 years (all *P* > 0.05). Women made up 21.3% of the PNV eyes, 30.1% of the PCV eyes, 30.8% of the typical AMD eyes, and 55.3% of the control eyes.

**Table 1:** Demographic of patients with pachychoroid neovasculopathy (PNV), polypoidal choroidal vasculopathy (PCV), typical age-related macular degeneration (typical AMD), and control.

Baseline Characteristics, n (eyes)	PNV	nAMD without pachyvessels n = 145		Control
		PCV	Typical AMD	
	n = 75	n = 93	n = 52	n = 150
Age (years)	71.3 ± 1.2	74.3 ± 0.9	75.7 ± 1.2	73.9 ± 0.6
Female sex, n (%)	16 (21.3)	28 (30.1)	16 (30.8)	83 (55.3)
BCVA (logMAR units)	0.4 ± 0.04	0.5 ± 0.05	0.6 ± 0.07	
Drusen, n (%)	17 (22.7)	15 (16.1)	14 (26.9)	
Subfoveal choroidal thickness (μm)	268.6 ± 4.9	214.8 ± 5.5	197.5 ± 7.9	
Choroidal vessel diameter (μm)	178.9 ± 2.9	98.0 ± 2.6	85.2 ± 3.7	

PNV, pachychoroid neovasculopathy; PCV, polypoidal choroidal vasculopathy; nAMD, neovascular age-related macular degeneration; typical AMD, typical age-related macular degeneration; logMAR, logarithm of minimum angle of resolution. Values are means ± standard error of the means. PNV, n = 75; nAMD without pachyvessels, n = 145; PCV, n = 93; typical AMD, n = 52; control, n = 150.

Drusen (non-extensive drusen or hard drusen)^14^ was detected in 22.7% of PNV eyes, 16.1% of the PCV eyes, and 26.9% of the typical AMD eyes. Polypoidal lesions were found in 56 eyes of the PNV patients (74.7%).

Because the PNV spectrum of diseases is characterized by a dilation of the choroidal vessels, we first compared the diameter of the choroidal vessels and the thickness of the choroid in the PNV, PCV, and typical AMD eyes. The PNV eyes had significantly thicker choroids and larger choroidal vessels than that of the PCV (*P* < 0.001) or typical AMD (*P* < 0.001) eyes (Table 1).

**Figure 1:**
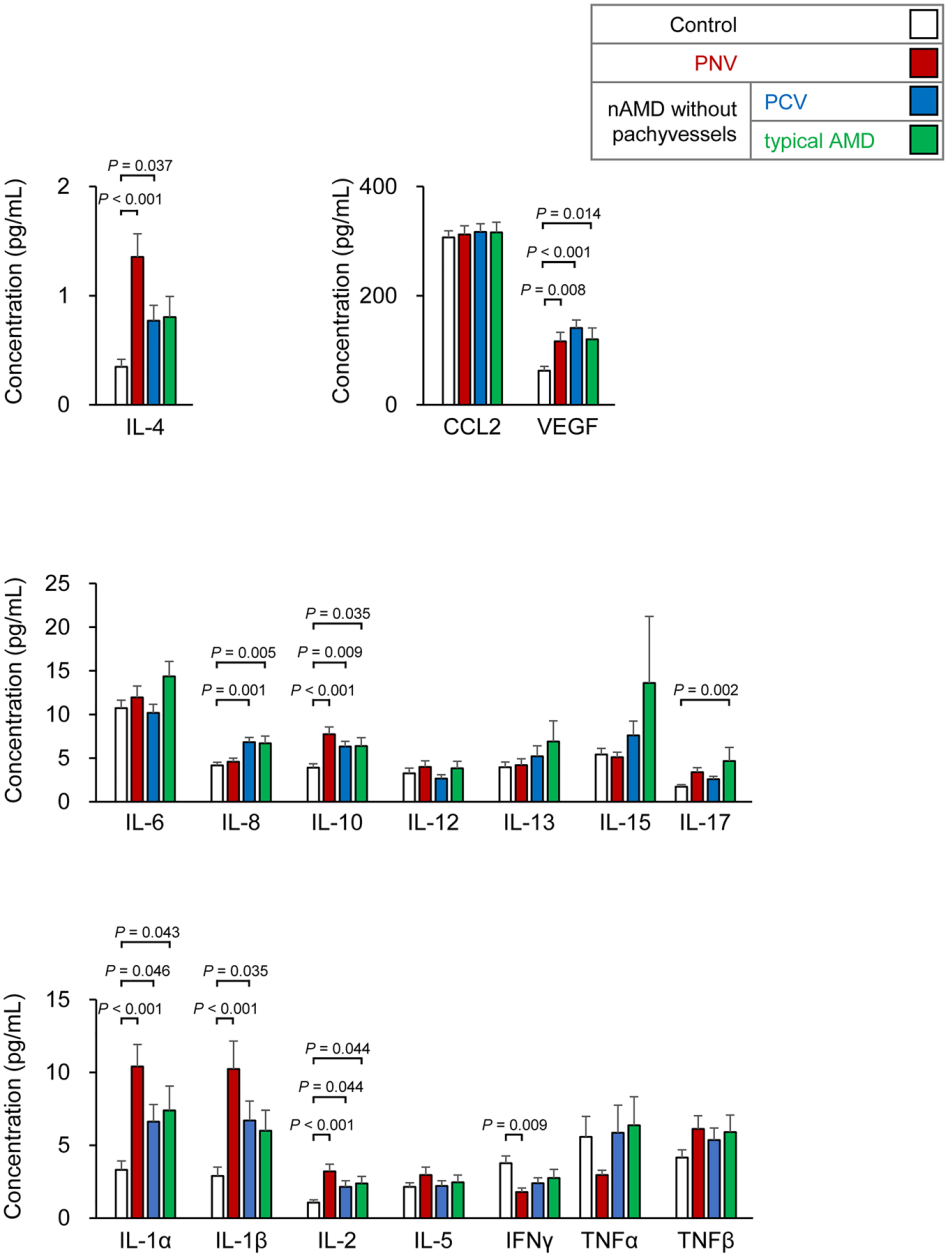
Levels of cytokines in the aqueous humor of eyes with pachychoroid neovasculopathy (PNV), polypoidal choroidal vasculopathy (PCV), typical age-related macular degeneration (typical AMD), and normal subjects. In PNV (red bar), the level of IL-1α, IL-1β, IL-2, IL-4, IL-10, and VEGF were significantly higher than that of the control eyes (white bar). In the PCV eyes (blue bar), IL-1α, IL-1β, IL-2, IL-4, IL-8, IL-10, and VEGF were significantly higher than that of the control eyes. The levels of IL-1α, IL-2, IL-8, IL-10, IL-17, and VEGF were higher in eyes with typical age-related macular degeneration (AMD; green bar) than in the normal control eyes. PNV, pachychoroid neovasculopathy; PCV, polypoidal choroidal vasculopathy; typical AMD, typical age-related macular degeneration. Data are the means (pg/ml) and standard error of the means. PNV, n = 75; PCV, n = 93; typical AMD, n = 52; control, n = 150. ANOVA and post hoc test (Dunnett).

We then assessed the levels of 17 intracameral cytokines in eyes with PNV, PCV, and typical AMD (Fig. 1). In the PNV eyes, the levels of IL-1α, IL-1β, IL-2, IL-4, IL-10, and VEGF were significantly higher than that of the control eyes. In the PCV eyes, the levels of IL-1α, IL-1β, IL-2, IL-4, IL-8, IL-10, and VEGF were significantly higher than the control eyes, and in the typical AMD eyes, the levels of IL-1α, IL-2, IL-8, IL-10, IL-17, and VEGF were also significantly higher.

These findings suggested that eyes with PNV have overlapping levels of cytokines with eyes with typical AMD and PCV although different levels of elevations were observed.

Next, we examined how each cytokine was associated with the PNV entity using logistic regression analyses. The associations with PNV were highest for IL-4 (/quintile, odds ratio (OR): 1.61, *P* < 0.001), followed by IL-1α (/quintile, OR: 1.32, *P* = 0.03), and IL-2 (/quintile, OR: 1.29, *P* = 0.04) after age adjustments.

### Association of subfoveal choroidal thickness and levels of inflammatory cytokines in pachychoroid neovasculopathy (PNV) eyes

The choroidal vessels in eyes with PNV are characterized by dilation, i.e., pachyvessels, and hyperpermeability which increase the choroidal thickness. Therefore, we next determined whether the levels of cytokines were significantly correlated with the choroidal thickness. In PNV, the highest correlation between the choroidal thickness and the different cytokines was observed for IL-4 (ρ = 0.36, *P* = 0.002), followed by IL-13 (ρ = 0.33, *P* = 0. 004), IL-12 (ρ = 0.33, *P* = 0.004), TNFα (ρ = 0.30, *P* = 0.008), IL-1β (ρ = 0.27, *P* = 0.02), IL-17 (ρ = 0.27, *P* = 0.02), IL-10 (ρ = 0.27, *P* = 0.02), IL-15 (ρ = 0.26, *P* = 0.03), and IL-1α (ρ = 0.24, *P* = 0.03; n = 75; Spearman correlation analysis).

The highest correlation between the choroidal vessel diameter and the levels of the different cytokines was found for IL-10 (ρ = 0.46, *P* < 0.001), followed by IL-4 (ρ = 0.32, *P* = 0.005), IL-12 (ρ = 0.30, *P* = 0.009), IL-5 (ρ = 0.29, *P* = 0.012), IL-1α (ρ = 0.29, *P* = 0.013), TNFα (ρ = 0.28, *P* = 0.016), IL-2 (ρ = 0.25, *P* = 0.03), TNFβ (ρ = 0.24, *P* = 0.03), and IL-13 (ρ = 0.24, *P* = 0.04; n = 75, Spearman correlation analysis).

Because the development of the pachyvessels may be associated with vascular remodeling, we also examined the associations between the levels of cytokines and number of polypoidal lesions. When PNV eyes with polypoidal lesions were assessed, the level of IL-4 (ρ = 0.48, *P* = 0.0002), IL-17 (ρ = 0.46, *P* = 0.0003), and TNFβ (ρ = 0.31, *P* = 0.02) were significantly correlated with the number of polypoidal lesions (n = 56, Spearman correlation analysis).

Thus, in PNV eyes, choroidal thickness, choroidal vessel diameter, and number of polypoidal lesions were associated with elevated levels of inflammatory cytokines.

To calculate direct effect of the inflammatory cytokines on choroidal thickness and choroidal vessel diameter, multivariate linear regression analyses were conducted after adjustments of the number of polypoidal lesions. The results indicated that in eyes with PNV, the elevations of the levels of IL-4, TNFα, IL-17, IL-2, IL-12, IL-15, IL-5, IL-13, IL-1α, and IL-1β were significantly correlated with an increased choroidal thickness (Table 2). Similarly, the levels of IL-4, IL-2, IL-5, IL-10, IL-13, TNFβ, IL-1α, and IL-1β were significantly correlated with larger choroidal vessel diameter (Table 3). Of all these cytokines, IL-4 had the highest contribution with the increase of 11.6 μm in the choroidal thickness and 6.72 μm in the choroidal vessel diameter by its unit increase.

**Table 2:** Association of inflammatory cytokines with subfoveal choroidal thickness in pachychoroid neovasculopathy (PNV).

Cytokine (pg/ml)	Coefficients	Standard error	*P*-value
IL-1α	1.66	0.52	0.002
IL-1β	1.18	0.39	0.003
IL-2	4.50	1.60	0.006
IL-4	11.62	3.95	0.004
IL-5	3.78	1.42	0.010
IL-12	4.32	1.18	< 0.001
IL-13	3.28	1.00	0.001
IL-15	4.18	1.41	0.004
IL-17	4.77	1.60	0.004
TNFα	6.75	2.43	0.007
CCL2	− 0.11	0.05	0.039

PNV, pachychoroid neovasculopathy. Multivariate linear regression analyses of subfoveal choroidal thickness after age, gender, and number of polypoidal lesions adjustment in PNV. PNV, n = 75.

**Table 3:** Association of inflammatory cytokines with choroidal vessel diameter in pachychoroid neovasculopathy (PNV).

Cytokine (pg/ml)	Coefficients	Standard error	*P*-value
IL-1α	0.89	0.30	0.005
IL-1β	0.55	0.24	0.022
IL-2	3.01	0.93	0.002
IL-4	6.72	2.23	0.005
IL-5	2.17	0.79	0.008
IL-10	1.82	0.56	0.002
IL-13	1.43	0.61	0.021
TNFβ	1.40	0.56	0.016

PNV, pachychoroid neovasculopathy. Multivariate linear regression analyses of subfoveal choroidal thickness after age, sex, and number of polypoidal lesions adjustment in PNV. PNV, n = 75.

### Modeling of association of choroidal thickness, choroidal pathological findings, and inflammatory cytokines

**Figure 2:**
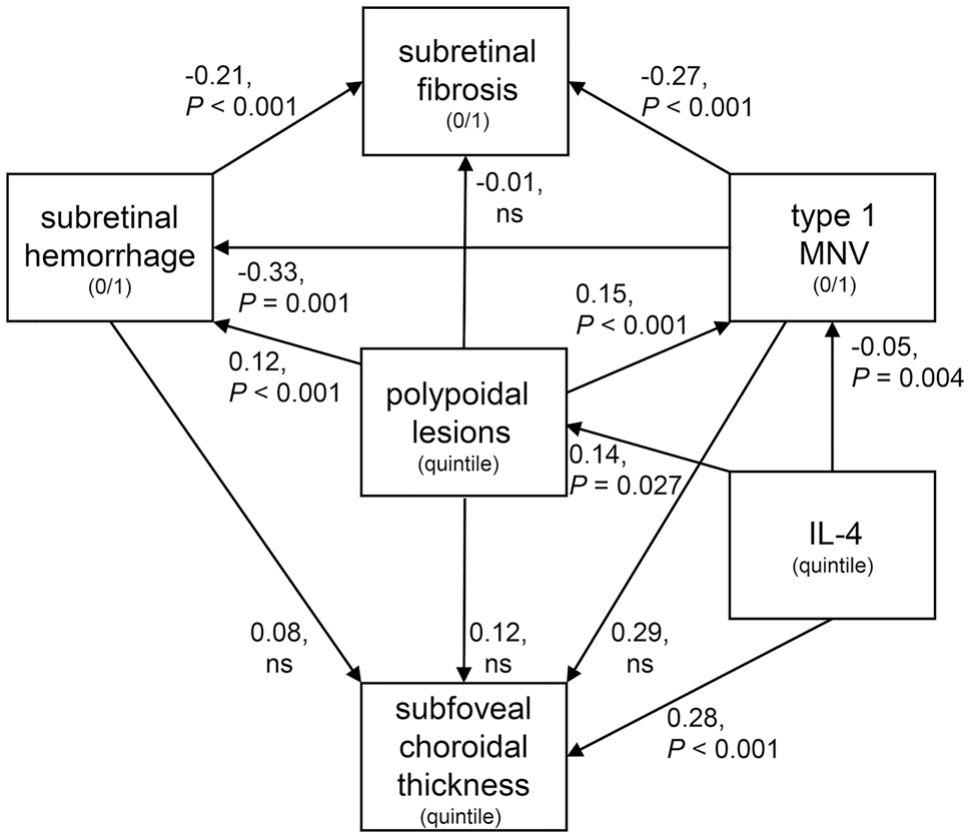
Structural equation modeling path diagrams for associations among IL-4 level and disease characteristics. Associated cytokines and pathological choroidal features associated with pachychoroid neovasculopathy and neovascular age-related macular degeneration are shown in the path diagram using structural equation modeling. Subretinal hemorrhage, subretinal fibrosis, and type 1 MNV were coded as 0/1. IL-4, subfoveal thickness, and polypoidal lesions were coded as 0/1/2/3 based on quintile. Structural equation modeling construction with the inclusion of clinical characteristics was conducted by assessment using fitting indices, Akaike information criteria (AIC), and Bayesian information criteria (BIC). Arrows are shown with coefficients of association and *P*-values in the path diagram. Comparative fit index: 1.000, root mean square error of approximation: < 0.001, MNV: macular neovascularization, polypoidal lesions: numbers of polypoidal lesions at the macular lesion. ns, not significant, n = 220.

The findings indicated that IL-4 and inflammatory cytokines had a significant effect on the choroidal thickness and the remodeling of the choroidal vasculature. Therefore, we examined how they contributed to the clinical characteristics including the choroidal thickness, polypoidal lesions, type 1 macular neovascularization (MNV), subretinal fibrosis, and subretinal hemorrhage. We first analyzed the associations of all the clinical characteristics using a structural equation modeling. After the construction of a model of the clinical characteristics (Fig. 2), the involvement of the significantly elevated cytokines was assessed. We found that IL-4 showed the highest significant association with the development of the clinical characteristics including the choroidal thickness, and polypoidal lesions.

A path diagram indicated that the IL-4/quintile significantly increased the choroidal thickness by 0.28 quintile (*P* < 0.001) as well as the vessel diameter by 0.30 quintile (*P* < 0.001) (Supplementary Figure S1). The effects of IL-4 on the number of polypoidal lesions was almost one-half of that on the choroidal thickness.

Interestingly, IL-4 had no direct association with the presence of subretinal hemorrhages or subretinal fibrosis. The number of polypoidal lesions/quintile was significantly associated with the presence of subretinal hemorrhages (*P* < 0.001).

### Association of IL-4 polymorphism and choroidal thickness

To examine effects of IL-4 on choroidal thickness in more detail, patients were assessed for *IL-4* polymorphism. When the common *IL-4 -590* polymorphism (rs2243250) was assessed, the level of T allele was significantly correlated with the subfoveal choroidal thickness (ρ = 0.42, *P* = 0.0012; n = 53) and the choroidal vessel diameter (ρ = 0.50, *P* = 0.0001; n = 53; Spearman correlation analysis).

## Discussion

The pachychoroid spectrum of diseases including PNV is a recently proposed disease entity characterized by pachyvessels^1,3^. Our results showed that IL-4 was associated with the presence of pachyvessels in the choroid and with the PNV spectrum of diseases. More specifically, the remodeling of the pachyvessels was associated with higher levels of IL-4 expression, which was partly associated with the formation of the polypoidal lesions. These findings suggested that IL-4 was associated with the vascular remodeling of the choroidal vessels and may play different roles in the PNV disease spectrum.

In the PNV disease spectrum, IL-4 was specifically associated with the choroidal thickness and blood vessel diameter, and both were associated with a single nucleotide polymorphism, *IL-4 -590* (rs2243250). This nucleotide is a well-recognized allele associated with asthma^15^ and rhinitis^16^, and its presence leads to an overexpression of the mRNA of IL-4^17^ and increased levels of plasma IL-4^18^.

Earlier, the molecular pathology of nAMD was examined by analyzing the aqueous humor of the patients. Several studies found a significant association of inflammatory cytokines with nAMD. For example, VEGF^19,20^, CCL2^5,6,21,22^, CCL3^22^, CCL4^22^, IL-1α ^23^, IL-6^7–9^, IL-8^7,9,10^, IL-15^23^, IL-36β^24^, TGF-β^21^, CXCL9 (C-X-C Motif Chemokine Ligand 9)^21^, CXCL10^23^, CXCL12^25^, angiopoietin-2^10^, HGF (hepatocyte growth factor)^10^, TIMP1 (tissue inhibitor of metallopeptidase 1)^10^, FGF1 (fibroblast growth factor 1)^24^, and angiogenin^24^ were found to be significantly associated with nAMD.

In contrast, there have been few reports on the associations of PNV. Earlier studies showed that PNV was associated with elevated levels of VEGF^6^, CCL2 (C–C motif chemokine 2)^6,25^, IL-8, angiopoietin-2, PGF (placental growth factor), CXCL12 (C-X-C Motif Chemokine Ligand 12), and CXCL13 (C-X-C Motif Chemokine Ligand 13)^25^. Thus, eyes with PNV have an overlapping profile of cytokine elevations with nAMD, however it is still unclear whether PNV has a specific cytokine profile.

PNV is characterized by the presence of pachyvessels, vascular hyperpermeability, and vasodilation of the outer choroidal vessels. Thus, the vascular hyperpermeability of the pachyvessels causes choroidal thickening and subretinal exudative changes^10^. However, previous reports on PNV assessed its association with the disease category, and no detailed analyses for the disease characteristics have been published. Thus, it remains unclear how the observed cytokines may be associated with or contribute to the formation of pachyvessels or vascular remodeling.

The critical disease-associated cytokines for PNV appeared to be VEGF based on the previous information on nAMD. However, in PNV, the contribution of the VEGF in the aqueous to PNV is inconsistent depending on the study. Kato et al. reported an elevation of VEGF in PNV eyes^6^, however the most recent study revealed that VEGF was significantly decreased in the eyes without drusen or decreased in eyes with non-pachychoroid, non-drusen, and it is likely that the presence of drusen can be associate with the VEGF level^25^. The same report showed that an elevation of CRP, CXCL12, CXCL13, IL-8, angiopoietin-2, PIGF, and CCL2 was present in eyes with PNV^25^. Although the involvement of various types of drusen on cytokine kinetics has not been fully analyzed, an elevation of IL-8 and CCL2 was not observed in PNV eyes in our cohort.

Because CCL2 is well recognized to be associated with nAMD, we also examined the association of CCL2 with choroidal thickness by structural equation modeling analyses. We found a significant and negative effect of CCL2 on the choroidal thickness (coefficient: -0.18, *P* = 0.007). However, the inclusion of CCL2 into the structural equation modeling analysis did not significantly change the structure of the outcomes. Thus, the role of CCL2 has not been determined and presumably may be context dependent.

T-helper 2 (Th2) lymphocytes and related cytokines including IL-4, IL-5, IL-6, IL-9, IL-13, and IL-17E (IL-25) play significant roles in the increased vascular permeability and vasodilatability. Of these, IL-4 decreases the endothelial barrier function which causes the vascular hyperpermeability^26,27^. Importantly, the IL-4-mediated hyperpermeability of the vascular endothelial cells has been shown to be a major mechanism for inflammatory edema^28^.

Mechanistically, IL-4 impairs the barrier function of the vascular endothelium by forming gaps between the endothelial cells through the rearrangement of the cytoskeleton^29^. Another mechanism for the IL-4 mediated barrier dysfunction has been recently reported. IL-4 stimulates the formation of actin stress fibers by a non-canonical Wnt ligand, Wnt5A^30^. This eventually causes a remodeling of the cytoskeleton leading to an impairment of the barrier function^30^. Thus, we propose that inflammatory networks featuring IL-4 contributes to vascular hyperpermeability leading to the development of the pachyvessels.

Another characteristic of pachyvessels is the vasodilation of the outer choroidal vessels. Vasodilation has also been reported to be associated with IL-4. IL-4 promotes both the lumen formation^13,31^ and the endothelial cell cycle^32^. In addition, IL-4 stimulates the VCAM-1 promoter via STAT6^33^ and exerts proangiogenic effects on the VCAM-1/alpha4 integrin pathway^31^. In addition, the vasodilation itself induces chronic ischemia and the hypoxic condition of the choriocapillaris^2^ leads to the induction of IL-4^34^.

Considering the pathogenic mechanism of PNV to be a venous overload choroidopathy, PNV is similar to diseases with chronic venous congestions^3^. A wide variety of venous disorders throughout the body including varicose veins^35^, pregnancy^36,37^, pelvic congestion syndrome^38^, and gastrointestinal varices associated with cirrhosis and portal hypertension^39^ are characterized by vasculopathies due to venous overload. The venous overload promotes capillary dilation and increases the permeability, inflammatory cell infiltration, activation of matrix metalloproteinases (MMPs)^40^, and the induction of cell adhesion molecules (CAMs)^41^.

Importantly, IL-4 has been shown to be associated with the development of vasodilatability, hypervascular permeability, and tissue vascular remodeling which is exemplified in the placenta during pregnancy^42^ and the regeneration of cardiomyocytes^43^.

Analysis of the aqueous humor showed that IL-4 was also associated with the polypoidal lesions^11^. In addition, elevated IL-4 levels may induce polypoidal formation or choroidal neovascularizations^13^. Polypoidal lesions are often accompanied by pachyvessels^44^. Because large polypoidal lesions can be regarded as neovascular tangles or branching neovascular networks rather than actual polypoidal lesions or aneurysmal dilatations in OCT images^45^, polypoidal lesions may reflect angiogenic lesions^44,46^. Our results showed that IL-4 was significantly associated with the choroidal thickness and the presence of polypoidal lesions in eyes with PNV. This is consistent with reports that showed that the development of polypoidal lesions was closely associated with development of pachyvessels^47^. Collectively, the PNV diseases appeared to be associated with vascular remodeling including the development of polypoidal lesions. In addition, the pachyvessels may also promote choroidal neovascularizations^48^.

There are some limitations in this study. Our study was a retrospective analysis and may not have a direct causal relationship. For example, there is a possibility that the contribution of IL-4 may be indirect effects of unobserved factors or molecules. However, our previous analyses using IL-4 deficient mice supported the significant and direct roles of IL-4 in vascular remodeling which supports our conclusions^13^. To understand the roles of IL-4 in greater detail, clinical trials of blocking IL-4 in PNV patients are required. Nevertheless, our study provides a strong basis for future therapy blocking the IL-4 pathway to treat patients with PNV spectrum of diseases.

In conclusion, our findings indicate that IL-4 represents a crucial mediator in the development of the PNV spectrum of diseases as well as the vascular remodeling in PNV patients.

## Methods

### Diagnosis and eligibility criteria of patients

All of the consecutive cases of PNV and nAMD that received intravitreal ranibizumab or aflibercept at the University of Tottori Hospital between 2013 January and 2018 September were studied.

Those who met the criteria for PNV were designated as PNV and the others as nAMD. We used the following criteria for the diagnosis of PNV which was based on earlier reports ^1,8,49,50^: (1) presence of type 1 macular neovascularization (MNV) detected in either eye; (2) choroidal vascular hyperpermeability detected in the late phase of indocyanine green angiography (ICGA); (3) presence of dilated choroidal vessels below type 1 MNV detected by optical coherence tomography (OCT); (4) no or only nonextensive drusen or hard drusen in both eyes (The Age-Related Eye Disease Study (AREDS) Report Number 6: Level 1)^14^.

**Figure 3:**
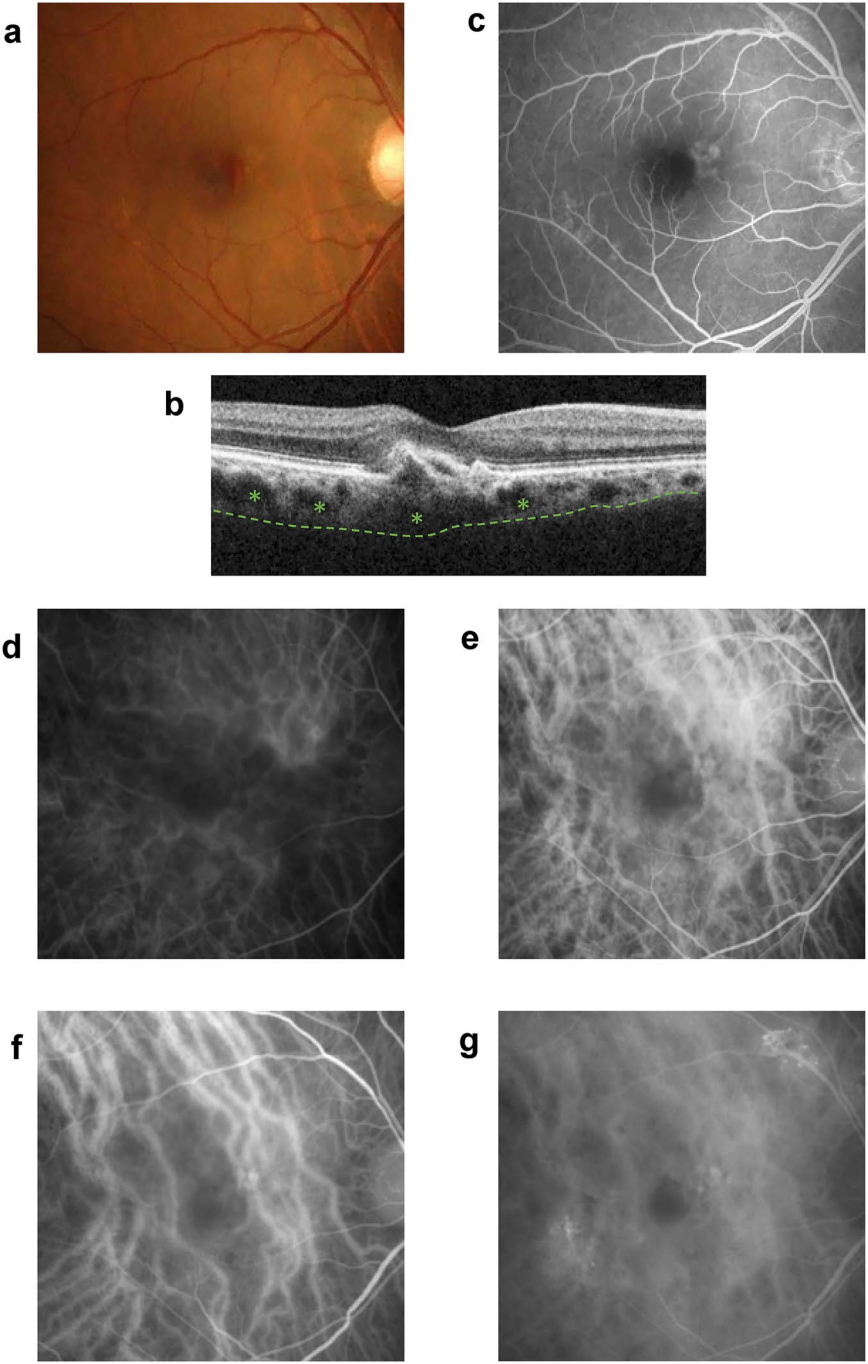
Representative case of pachychoroid neovasculopathy. (**a**) Fundus photograph of a 62-year-old female patient showing a retinal pigment epithelium (RPE) abnormality and a subretinal hemorrhage in the macular area. Large choroidal vessels can be seen on the nasal and temporal side of the macular area. (**b**) OCT image showing choroidal thickening, dilated choroidal pachyvessels with inner choroidal attenuation (asterisks). The dotted line represents the choroid-sclera interface. An RPE detachment can be seen at the fovea. (**c**) Fluorescein angiogram showing window defects and some discharge in the macular area in the early phase (22 s). (**d**)–(**g**) Indocyanine green angiogram showing dilated choroidal vessels with focal choroidal hyperpermeability and macular neovascularization (MNV) with polypoidal lesions at the macular area. (d; 14 s, e; 17 s, f; 29 s, g; 10 min 28 s).

A representative case of typical PNV is shown in Fig. 3. This was a 62-year-old female patient with polypoidal lesions who was diagnosed as having PNV. A retinal pigment epithelium (RPE) abnormality and leaky large choroidal vessels were observed in the ICGA.

PCV was diagnosed in the presence of a branching vascular network with terminal aneurysmal dilatations in the ICGA images corresponding to an RPE elevation in the OCT images^44^.

Typical AMD was diagnosed by the presence of MNV and other findings corresponding to the AREDS Report Number 6, Levels 2, 3, and 4^8,14^ (Supplementary Figure S2).

The exclusion criteria were eyes with retinal angiomatous proliferation (RAP), MNVs secondary to high myopia with a refractive error (spherical equivalent) ≤  − 6.00 D, trauma, angioid streaks, uveitis, any other neovascular maculopathy, prior laser photocoagulation, photodynamic therapy, and intraocular surgery within the past 3 months.

All the patients underwent a unified diagnosis by three independent examiners (A.K., R.U., and H.M.). If the three diagnoses did not match, the senior investigators (K.I. and S.S.) made the final decision after discussions with other retina specialists.

The *IL-4* polymorphism was examined in 14 PNV and 39 nAMD patients without pachyvessels. All procedures were performed with consent from the patient. For the control groups, aqueous humor was collected from 150 eyes of 150 normal patients who underwent routine cataract surgery.

The procedures used in this study were approved by the institutional Review Board of the University of Tottori, and they conformed to the tenets of the Declaration of Helsinki. A signed written informed consent was obtained from all participants.

### Measurement of cytokines in aqueous humor

On the day of the initial anti-vascular endothelial growth factor (anti-VEGF) treatment, 100 μL of aqueous humor was collected by paracentesis before the injection of the anti-VEGF agent and stored at − 80 °C until cytokine measurements.

Aqueous humor samples were analyzed for the levels of IL-1α, IL-1β, IL-2, IL-4, IL-5, IL-6, IL-8, IL-10, IL-12, IL-13, IL-15, IL-17, IFN-γ, TNF-α, TNF-β, VEGF, and CCL2 using commercially available ELISA kits (Quansys Biosciences, West Logan, UT), PeproTech (Rocky Hill, NJ) for CCL2, and R&D Systems (Minneapolis, MN) for VEGF^11,51^.

### Genetic analyses

Genomic DNA was obtained from peripheral blood using a standard protocol.

Sequencing was performed with the BigDye Terminator v3.1 Cycle Sequencing Kit (Thermo Fisher Scientific Inc., Waltham, MA) and a 3500xL Genetic Analyzer capillary sequencer (Thermo Fisher Scientific Inc.) as described^52^. All of the primer sequences are available on request.

### Image analyses and phenotyping

All patients underwent comprehensive ophthalmic examinations including fluorescein angiography (FA), indocyanine green angiography (ICGA), and swept-source optical coherence tomography (OCT) using DRI OCT Triton (Topcon Corporation, Tokyo).

The subfoveal choroidal thickness was measured from the retinal pigment epithelium to the choroid-sclera interface at the foveal center. The choroidal vessel diameter (vertical diameter of the thickest outer choroidal vessel in the foveal region) was measured by referring to the scale bars in the OCT system.

### Statistical analyses

The decimal best-corrected visual acuity (BCVA) was measured with a Landolt chart and converted to the logarithm of minimum angle of resolution (logMAR) for the statistical analyses. Logistic regression analyses were performed to calculate the odds ratios (OR) based on quintiles of each cytokine level. Each cytokine quintile was compared with the lowest quintile as the reference category. Unpaired *t* tests, Mann–Whitney U tests, and ANOVA with post hoc analysis were used to determine whether the differences between groups were statistically significant. Data are presented as means and standard error of the means.

For the logistic regression analyses and structural equation modeling, the clinical characteristics were coded as (0/1). The model was selected based on fit index (Akaike information criteria (AIC) and Bayesian information criteria (BIC))^53^.

The statistical analyses were performed using Stata 16.0 (StataCorp, College Station, TX). A *P*-value < 0.05 was considered statistically significant.

## Supplementary Information

Supplementary Information 1.

Supplementary Figure S1.

Supplementary Figure S2.

Supplementary Table S1.

## Data Availability

The known genetic polymorphism (rs2243250) analyzed during the current study is available in the dbSNP repository, [https://www.ncbi.nlm.nih.gov/snp/rs2243250]. The other datasets for the current study are available from the corresponding author on reasonable request.
