# Supplementary material for: Association of IL-4 with pachychoroid neovasculopathy
Source: Sci Rep. 2023 Jan 20;13:1152. doi: 10.1038/s41598-023-28108-y (PMC9860019; doi:10.1038/s41598-023-28108-y)
Supplement: Supplementary file 1 — Supplementary Information 1. [file 41598_2023_28108_MOESM1_ESM.docx]

**­Association of IL-4 with pachychoroid neovasculopathy**

Takashi Baba^1*^, Ayumi Koyama^1^, Ryu Uotani^1^, Hitomi Miyake^1^, Kodai Inata^1^, Shin-ichi Sasaki^1^, Yumiko Shimizu^1^, Yoshitsugu Inoue^1^, Kaori Adachi^2^, Eiji Nanba^2,3^, Dai Miyazaki^1^

^1^Division of Ophthalmology and Visual Science, Faculty of Medicine, Tottori University

^2^Research Initiative Center, Organization for Research Initiative and Promotion, Tottori University

^3^Otani Hospital

^*^Correspondence

Takashi Baba. (email: baba@tottori-u.ac.jp)

**Supplementary Table**

**Supplementary Table S1.** Demographic of medical history in patients with pachychoroid neovasculopathy (PNV), polypoidal choroidal vasculopathy (PCV), typical age-related macular degeneration (typical AMD), and control.

PNV, pachychoroid neovasculopathy; PCV, polypoidal choroidal vasculopathy; nAMD, neovascular age-related macular degeneration; typical AMD, typical age-related macular degeneration. Values are means ± standard error of the means. PNV, n = 75; nAMD without pachyvessels, n = 145; PCV, n = 93; typical AMD, n = 52; control, n = 150.

**Supplementary Figure legends**

**Supplementary Figure S1.** Structural equation modeling path diagrams for associations among IL-4 level and disease characteristics including choroidal vessel diameter.

Associated cytokines and pathological choroidal features associated with pachychoroid neovasculopathy and neovascular age-related macular degeneration are shown in the path diagram using structural equation modeling.

Subretinal hemorrhage, subretinal fibrosis, and type 1 MNV were coded as 0/1. IL-4, choroidal vessel diameter, and polypoidal lesions were coded as 0/1/2/3 based on quintile.

Structural equation modeling construction with the inclusion of clinical characteristics was conducted by assessment using fitting indices, Akaike information criteria (AIC), and Bayesian information criteria (BIC). Arrows are shown with coefficients of association and P values in the path diagram. Comparative fit index: 1.000, root mean square error of approximation: < 0.001, MNV: macular neovascularization, polypoidal lesions: numbers of polypoidal lesions at the macular lesion. ns, not significant, n = 220.

**Supplementary Figure S2.** Representative case of typical age-related macular degeneration.

**a:** Fundus photograph of a 76-year-old male patient showing a retinal pigment epithelium (RPE) abnormality and a subretinal hemorrhage in the macular area. Choroidal vessels can be seen on the temporal side of the macular area.

**b:** OCT image showing choroidal vessels with inner choroidal attenuation (asterisks). The dotted line represents the choroid-sclera interface. A macular neovascularization (MNV) and intraretinal fluid can be seen at the fovea.

**c:** Fluorescein angiogram showing leakage in the macular area in the late phase (53 s).

**d - f:** Indocyanine green angiogram showing choroidal vessels and neovascularization (MNV) at the macular area. A focal choroidal hyperpermeability was unclear compared to that in the pachychoroid neovasculopathy shown in Figure 3 (d; 21 s, e; 2 min 11 s, f; 14 min 04 s).
