# Supplementary figures and images for: Association of IL-4 with pachychoroid neovasculopathy
Source: Sci Rep. 2023 Jan 20;13:1152. doi: 10.1038/s41598-023-28108-y (PMC9860019; doi:10.1038/s41598-023-28108-y)

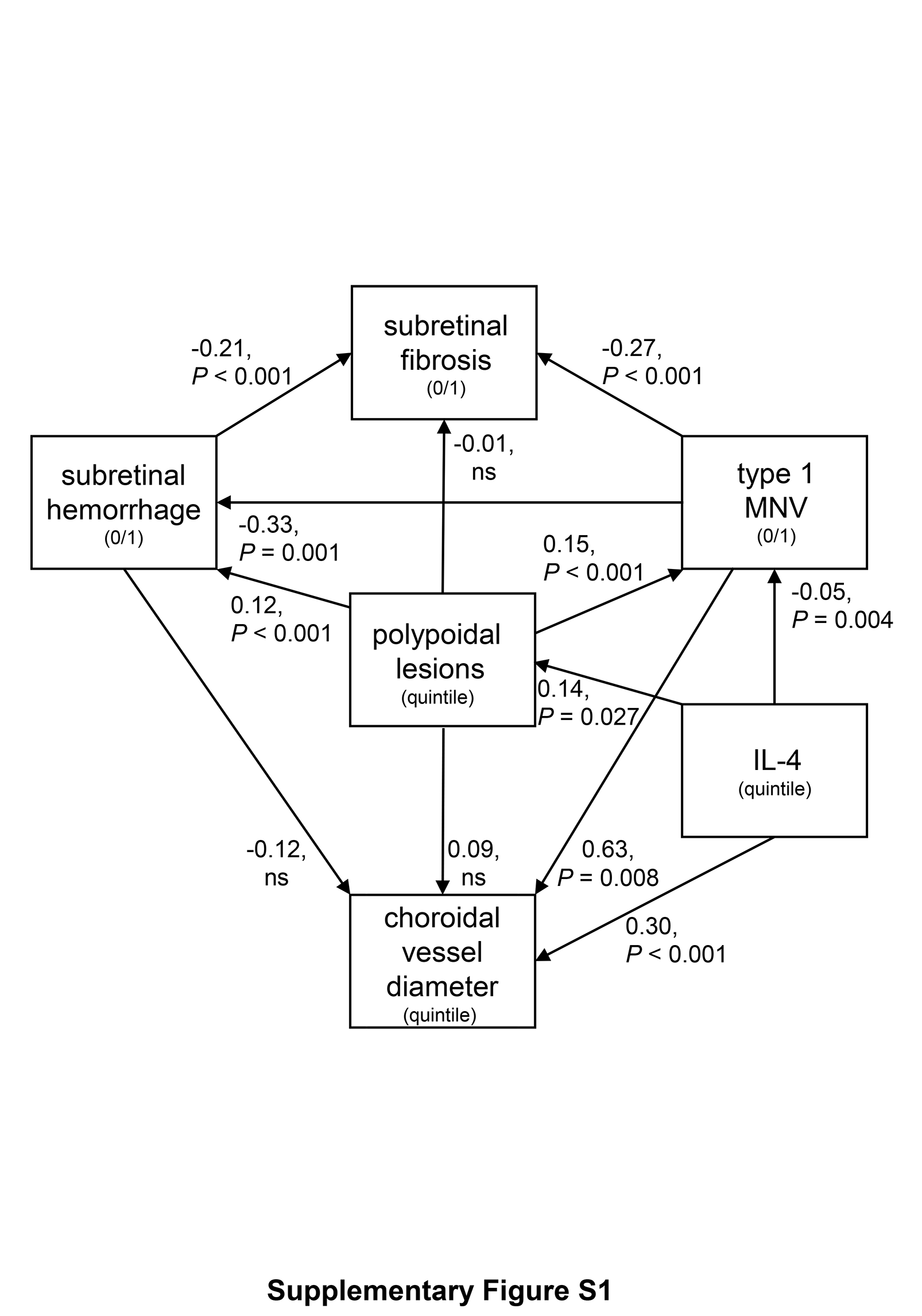

Supplement: Supplementary file 2 — Supplementary Figure S1. [file 41598_2023_28108_MOESM2_ESM.tif]

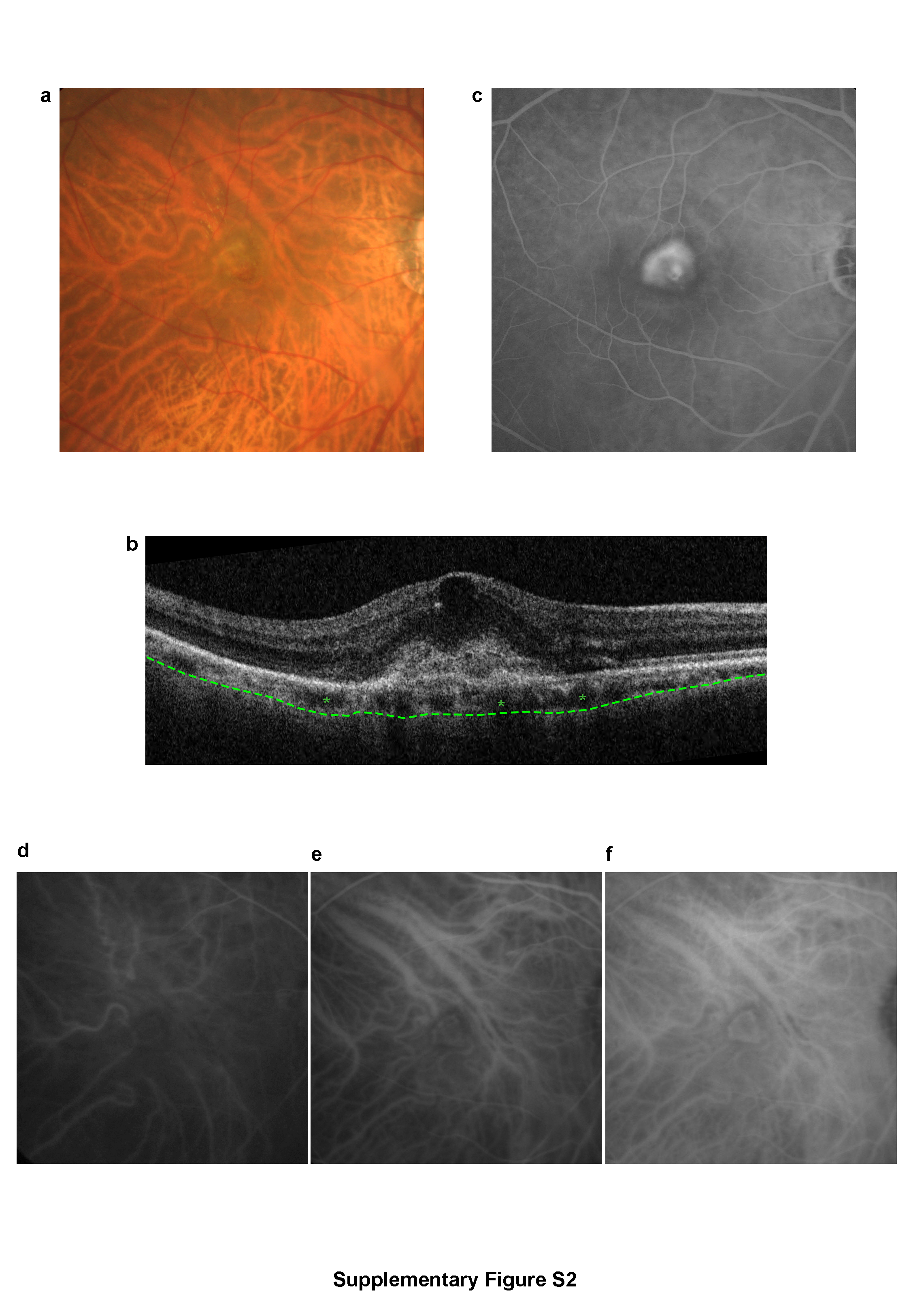

Supplement: Supplementary file 3 — Supplementary Figure S2. [file 41598_2023_28108_MOESM3_ESM.tif]
