# Supplementary material for: Association of IL-4 with pachychoroid neovasculopathy
Source: Sci Rep. 2023 Jan 20;13:1152. doi: 10.1038/s41598-023-28108-y (PMC9860019; doi:10.1038/s41598-023-28108-y)
Supplement: Supplementary file 4 — Supplementary Table S1. [file 41598_2023_28108_MOESM4_ESM.docx]

**Supplementary Table S1. Demographic of medical history in patients with pachychoroid neovasculopathy (PNV), polypoidal choroidal vasculopathy (PCV), typical age-related macular degeneration (typical AMD), and control**

| Baseline Characteristics, n (eyes) |  | nAMD without pachyvessels | |  |
| --- | --- | --- | --- | --- |
|  | PNV | n = 145 | | Control |
|  |  | PCV | Typical AMD |  |
|  | n = 75 | n = 93 | n = 52 | n = 150 |
| Hypertension, n (%) | 41 (54.7) | 47 (50.5) | 30 (57.7) | 63 (42.0) |
| Hyperlipidemia, n (%) | 23 (30.7) | 33 (35.5) | 12 (23.1) | 50 (33.3) |
| Diabetes, n (%) | 12 (16.0) | 15 (16.1) | 7 (13.5) | 25 (16.7) |
| Anticoagulation treatment, n (%) | 10 (13.3) | 21 (22.6) | 11 (21.2) | 34 (22.7) |
| Malignancy, n (%) | 15 (20.0) | 16 (17.2) | 10 (19.2) | 27 (18.0) |
| Allergic rhinitis, n (%) | 22 (29.3) | 20 (21.5) | 7 (13.5) | 25 (16.7) |
| Asthma, n (%) | 1 (1.3) | 2 (2.2) | 0 (0) | 2 (1.3) |
| Smoking, n (%) | 7 (9.3) | 5 (5.4) | 4 (7.7) | 2 (1.3) |

PNV, pachychoroid neovasculopathy; PCV, polypoidal choroidal vasculopathy; nAMD, neovascular age-related macular degeneration; typical AMD, typical age-related macular degeneration. Values are means ± standard error of the means. PNV, n = 75; nAMD without pachyvessels, n = 145; PCV, n = 93; typical AMD, n = 52; control, n = 150.
